# Quantitation of circulating DNA in the serum of breast cancer patients by real-time PCR

**DOI:** 10.1038/sj.bjc.6601609

**Published:** 2004-03-02

**Authors:** S Gal, C Fidler, Y M D Lo, M Taylor, C Han, J Moore, A L Harris, J S Wainscoat

**Affiliations:** 1 Nuffield Department of Clinical Laboratory Sciences, University of Oxford, John Radcliffe Hospital, Headington, Oxford OX3 9DU, UK; 2 Department of Chemical Pathology, The Chinese University of Hong-Kong, Prince of Wales Hospital, Shatin, New Territories, Hong-Kong SAR, China; 3 Cancer Research UK, Medical Oncology Unit, Churchill Hospital, Oxford, OX3 7LJ, UK; 4 Cancer Research UK, Molecular Oncology Laboratory, University of Oxford, Institute of Molecular Medicine, John Radcliffe Hospital, Headington, Oxford, OX3 9DS, UK

**Keywords:** serum, circulating DNA, breast cancer, real-time PCR

## Abstract

The purpose of this study was to quantify the level of serum DNA in different groups of primary breast cancer patients and in healthy controls using real-time quantitative PCR in order to determine whether such measurements have diagnostic or prognostic value. A total of 96 serum samples of patients with primary breast cancer before surgery (with positive or negative lymph nodes and with high or low relapse-free survival) as well as 24 healthy controls were analysed. DNA concentrations in the serum of the patients differed significantly from the concentration of serum DNA in the controls (medians were 221 and 63 ng ml^−1^, respectively, *P*<0.001 M–W test). However, no statistically significant difference was observed between the patient groups (*P*=0.87, M–W test). The serum DNA levels were elevated independently of the size of primary tumour or lymph node metastases. The overall survival of patients with serum DNA concentrations >221 ng ml^−1^ was better than patients with serum DNA concentration ⩽221 ng ml^−1^ (Kaplan–Meier, *P*=0.028).

Breast cancer is the most common malignancy in the female western population, responsible for up to one in five cancer-related deaths among women (Chen *et al*, 1999). Early detection and a reliable follow-up of breast cancer are crucial for successful treatment. For a definitive diagnosis, a tumour biopsy is required; however, a noninvasive test for early detection of the disease and for monitoring disease progression has been a goal for many researchers.

The presence of circulating nucleic acids in plasma and serum was first described many years ago (Mandel and Métais, 1947; Tan *et al*, 1966; Koffler *et al*, 1973). By using methods such as radioimmunoassay, it was discovered that cancer patients have higher levels of circulating DNA than those with nonmalignant diseases and healthy controls (Leon *et al*, 1977). Further more, it was shown that the level of circulating DNA is higher for patients with advanced disease (Shapiro *et al*, 1983).

In the last decade, with the development of more sensitive molecular methods such as the polymerase chain reaction (PCR), a renewed interest in circulating DNA has emerged. Many studies have been concerned with the detection of genetic changes in tumour DNA. A major effort has been devoted to the detection of mutations, loss of heterozygosity (LOH), microsatellites and the methylation status of DNA extracted from tumor tissue and plasma or serum of patients suffering from various tumours (Vasioukhin *et al*, 1994; Chen *et al*, 1996; Nawroz *et al*, 1996; Wong *et al*, 1999).

More recently, further studies have been carried out trying to determine if the level of circulating DNA can be used as a diagnostic or prognostic marker for cancer, as well as for other conditions such as pregnancy complications and trauma (Lo *et al*, 2000; Leung *et al*, 2001). For example, a DNA DipStick TM Kit was used to measure circulating DNA in the plasma of lung cancer patients (Sozzi *et al*, 2001), others have used spectrophotometry for the same purpose (Silva *et al*, 1999; Silva *et al*, 2002). In this study, we have used a real-time PCR method (Lo *et al*, 1998) to quantify the level of circulating DNA in controls and four different groups of patients diagnosed with breast caner. This method provides an accurate quantitation of the low concentrations of DNA in serum, enabling the results presented here to be directly compared to future studies. The present study is the first large study of serum DNA quantitation in breast cancer by real-time PCR.

## MATERIALS AND METHODS

### Patients and samples

We performed a retrospective study analysing serum samples from 96 women diagnosed with primary breast cancer collected before surgery. Four groups of patients were chosen: 26 cases had negative lymph nodes (LN) and low relapse-free survival (rfs⩽5 years) (group A); 22 cases had negative LN and high rfs (>5 years) (group B), 25 cases had positive LN and low rfs (group C) and 23 cases had positive LN and high rfs (group D). These groupings were chosen to analyse whether analysis of serum DNA would help to define the different risk groups independently of node status. A total of 24 frozen serum samples from healthy women blood donors were used as controls. Samples were selected on the basis of sufficient serum and appropriate clinical data available. All patients signed an informed consent form approved by the Oxford Radcliffe Hospitals ethical committee.

### Sample preparation

DNA was extracted from 400 *μ*l of serum using QIAamp DNA Blood Mini Kit (Qiagen, UK), according to the ‘blood and body fluid protocol’, with an elution volume of 50 *μ*l. DNA samples were frozen at −20°C until further processing.

### Real-time PCR

All PCR reactions were performed on an Applied Biosystems 5700 Sequence Detection System using the 5′nuclease assay. In the 5′nuclease assay-based real-time PCR, a fluorescent probe containing a reporter dye at the 5′ end and a quencher dye at the 3′ end is included in the reaction in addition to two primers. During the reaction, cleavage of the probe by the AmpliTaq Gold enzyme separates the reporter dye and the quencher dye, which results in increased fluorescence of the reporter. Accumulation of PCR products is detected directly by monitoring the increase in fluorescence by a charge-coupled device camera.

For each reaction, a threshold cycle or *C*_T_ value is determined. This value is the cycle at which a statistically significant increase in fluorescence is first detected. The higher the starting quantity of a target sequence, the earlier a significant increase in fluorescence is detected.

### Standard curve

By using a standard curve, it is possible to calculate the absolute concentration of target DNA in a sample. In this study, we generated a standard curve using five-fold serial dilutions of known concentrations of DNA (1000, 200, 40, 8, 1.28, 0.64 genome equivalents *μ*l^−1^).

### Polymerase chain reaction amplification

Serum DNA was measured using a real-time quantitative assay for the *β-globin* gene, according to the method developed by Lo *et al*. The assay included two primers, *β-globin*-345F, 5′-GTG CAC CTG ACT CCT GAG GAG A-3′; *β-globin*-445R, 5′-CCT TGA TAC CAA CCT GCC CAG-3′, and a dual-labelled fluorescent TaqMan probe, *β-globin*-402T, 5′-(FAM) AAG GTG AAC GTG GAT GAA GTT GGT GG(TAMARA)-3′. Sequence data were taken from the GeneBank Sequence Database accession no. U01317. Polymerase chain reaction was performed in a final volume of 25 *μ*l and contained 12.5 *μ*l of TaqMan 2 × Universal PCR Master Mix (AmpliTaq Gold® DNA polymerase, AmpErase® UNG, dNTPs with dUNTP, Passive reference 1 and optimised buffer components), 100 nM of Taqman probe, 300 nM of each primer and 4 *μ*l of extracted DNA.

Each sample was analysed in triplicate. Triplicates of the standard curve were included in each run.

### Data analysis

The mean quantity of each triplicate calculated by the 5700 sequence detection system software was used for further analysis. As described by Lo *et al* the concentration, expressed in copies per millilitre, was calculated using the following equation:


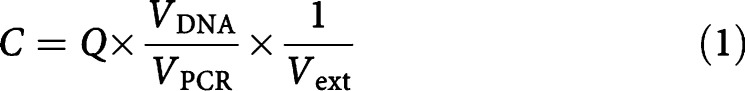


where *C*=target concentration in plasma (copies per millilitre); *Q*=target quantity (copies) calculated by the sequence detection system, *V*_DNA_=total volume of extraction (50 *μ*l); *V*_PCR_=volume of DNA solution used per PCR reaction (4 *μ*l); and *V*_ext_=volume of plasma extracted (400 *μ*l).

The concentration in ng ml^−1^ was calculated by using 6.6 pg of DNA per cell as a conversion factor.

### Statistical analysis

Data analysis was performed using StatView software and STATA analysis package.

## RESULTS

### The dynamic range of the real-time PCR

**Figure 1:**
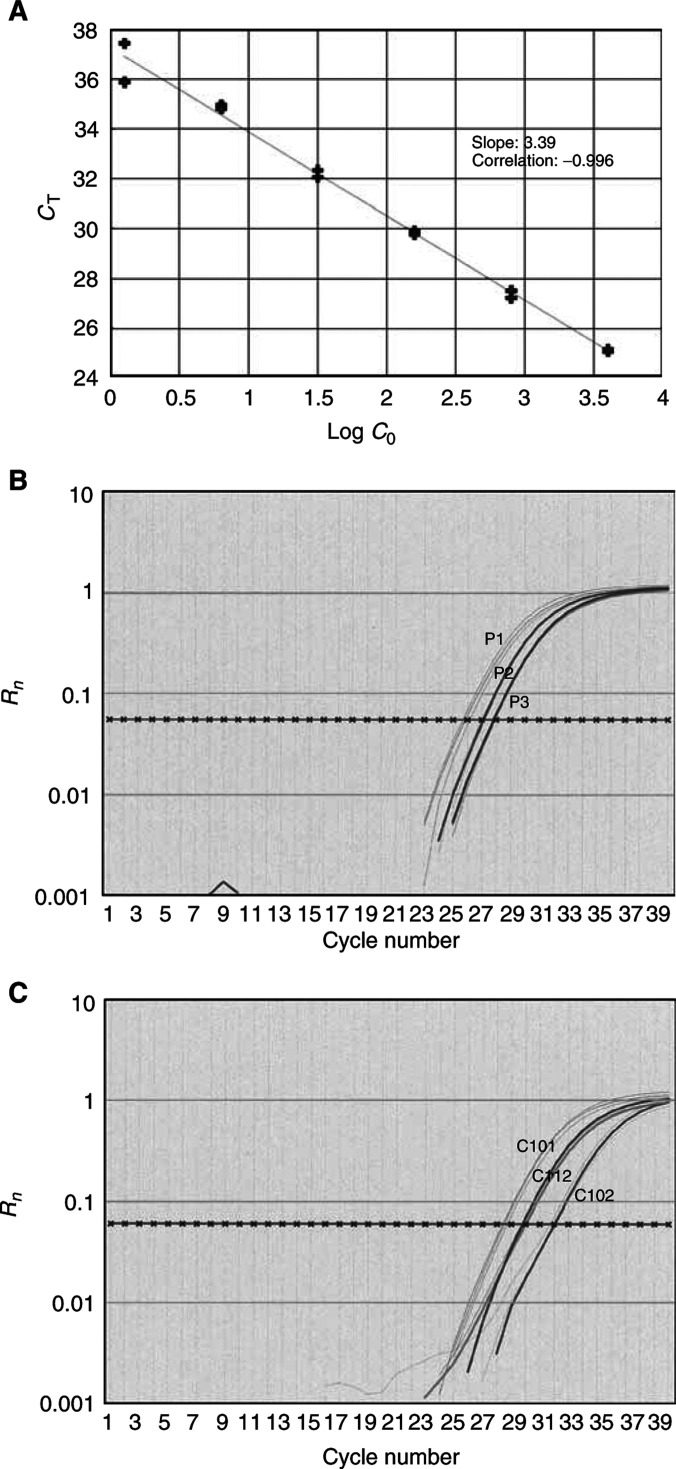
Real-time PCR. (**A**) A standard curve for the *β*-globin assay, using five-fold serial dilutions of genomic DNA (from 4000 to 1 genome equivalents). The *X*-axis denotes the threshold cycle (*C*_T_) and the *Y*-axis denotes the concentration of the target (logarithmic scale). (**B**) Amplification curves (triplicates) of three patients with breast cancer. (**C**) Amplification curves (triplicates) of three controls. The *X*-axis for (**B**) and (**C**) denotes the threshold cycle (*C*_T_) and the *Y*-axis denotes the *R*_n_, which is the fluorescence intensity.

The real-time PCR assay for *β-globin* enabled the detection of four orders of magnitude, from DNA equivalent of 4000 cells to a single cell. A strong linear relationship between the *C*_T_ values and the log of the copy numbers was observed (*R*^2^>0.99). All the serum sample concentrations fell within the values of the standard curve (Figure 1

).

### DNA concentrations in serum

The median of the serum DNA concentration for the controls was 63 (ng ml^−1^) (range 5–456 ng ml^−1^). The median of the serum DNA concentration for the patients was 221 ng ml^−1^ (range 17–3325 ng ml^−1^). The median values and ranges for the different groups of patients were as follows: 195 (27–2275) ng ml^−1^ for group A, 191 (24–3325) ng ml^−1^ for group B, 297 (17–1466) ng ml^−1^ for group C and 225 (24–2783) ng ml^−1^ for group D. The difference between the patients and the controls was statistically significant (Mann–Whitney rank sum test, *P*<0.001). There was no significant difference between the patient groups (Kruskal–Wallis test, *P*=0.87). The data are summarised in Table 1

**Table 1:** Serum DNA concentrations of the patients and controls

	**Number of patients**	**Median concentration of DNA (ng ml^−1^)**	**Range (ng ml^−1^)**	**Percentage (%) of cases with DNA concentration>100ng ml^−1^**	***P*-value**
Group A	26	195	27–2275	65%	*P*=0.87
Group B	22	191	24–3325	74%	(K–W test)
Group C	25	297	17–1466	72%	
Group D	23	225	24–2783	78%	

Groups A–D	96	221	17–3325	71.5%	*P*<0.001
Controls	24	63	5-456	12.5%	(M–W test)

K–W test=Kruskal–wallis test; M–W test=Mann–Whitney rank sum test.

and Figure 2

**Figure 2:**
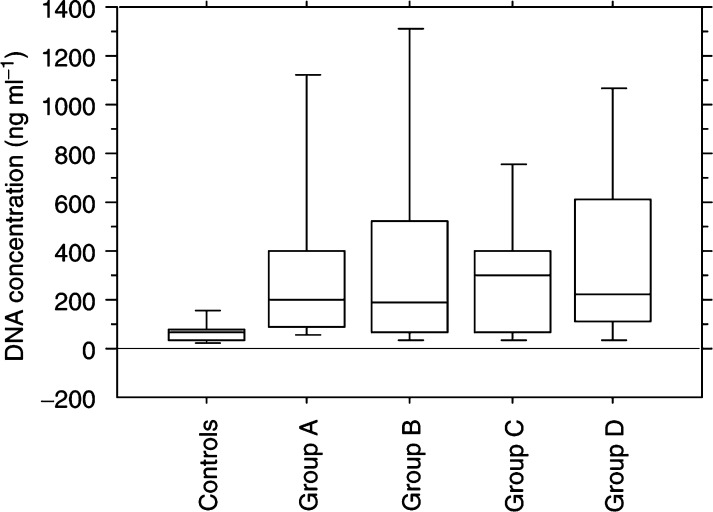
Box plot showing the concentrations of DNA in the serum of the different groups of patients and controls. The *X-*axi*s* shows the subject categories and the *Y-*axi*s* represents the concentration of serum DNA (ng ml^−1^). The upper and lower limits of the boxes indicate the 75th and 25th percentiles, respectively. The lines inside the boxes indicate the median. The upper and lower horizontal bars indicate the 90th and 10th percentiles, respectively.

**Figure 3:**
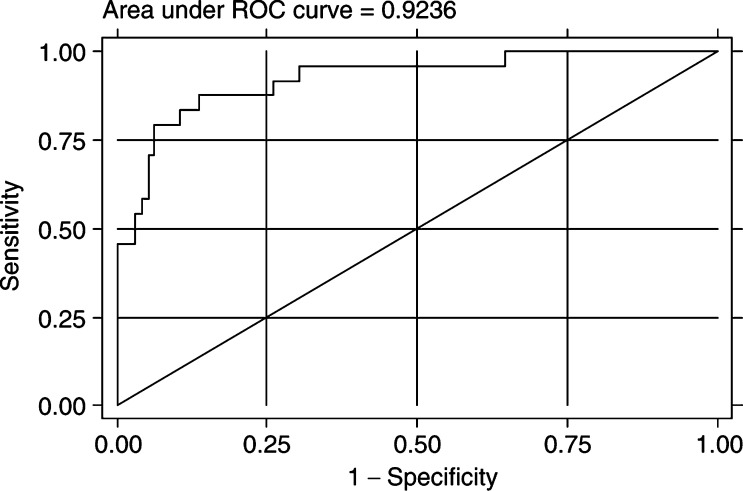
ROC curve analysis of serum DNA for the prediction of malignancy.

. Receptor operating characteristics (ROC) curve analysis was performed taking into account a difference in age between the patients and controls. The area under the ROC curve was 0.92. The sensitivity of the model was 70.8% and the specificity was 93.7% (Figure 3

).

Using a nonparametric rank sum to analyse the results and prognostic factors, we observed an inverse association of DNA concentration with age. The median for age⩾50 years (174 (17–2783) ng ml^−1^) was significantly lower than the median for age<50 years (319 (24–3325) ng ml^−1^), *P*=0.03. Analysing age as a continuous variable using Spearman's test for correlation also demonstrated a statistically significant but weak inverse correlation (*P*=0.01, rho=−0.26). No association with size (*P*=0.25), nodal status (*P*=0.68), oestrogen receptor (ER) (*P*=0.15) and epidermal growth factor receptor (EGFR) (*P*=0.35) was found. Table 2

**Table 2:** Biochemical and clinical characteristics used for the analysis

	**Number of patients**	**Median**	**Range**
*Nodal status*			
Negative	47		
Positive	49		
Size (cm)		2.2	0.1–10
⩽2	44		
>2	50		
			
*Histology*			
Infiltrating ductal	69		
Infiltrating lobular	15		
Infiltrating ductal/lobular	6		
Other infiltrating	6		

*ER status*		34	0.6–742
Negative, <10 fmols mg^−1^	25		
Positive, ⩾10 fmol mg^−1^	50		
EGFR status		23	0–241
Negative, <20	33		
Positive, ⩾20	40		

*Age*			
Patients		56	25–83
<50	30		
⩾50	66		
Controls	24	39	23–58

lists the characteristics overall of the population and the categories used for the analysis.

**Figure 4:**
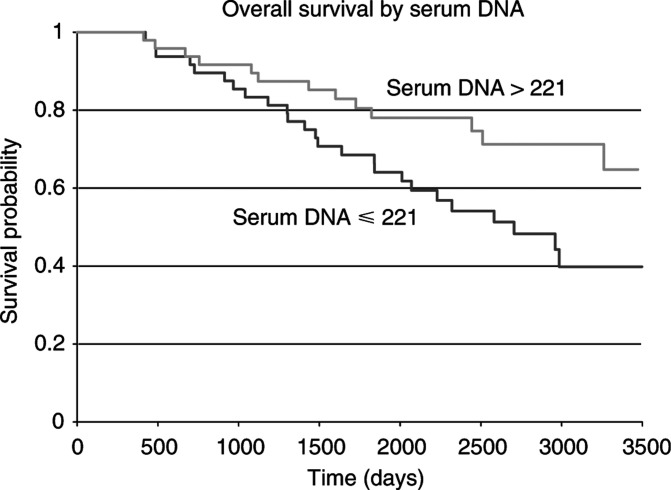
Overall survival (log-rank statistic) of the breast cancer patients according to serum DNA concentrations using the median concentration (221 ng ml^−1^) as a cut point (*P*=0.028).

We performed a univariate analysis of overall survival (OS) for known prognostic factors using the log-rank statistic. Tumour size and ER status were significantly associated with OS. Patients with larger tumours (>2 cm) did worse than patients with small tumours (⩽2 cm) (*P*=0.026), and ER-positive patients did better than ER-negative patients (*P*=0.009). No association was observed between OS and age (*P*=0.54) or EGFR status (*P*=0.63). We did not expect any relation of node status to OS because we selected node positive and negative groups with good and poor survival and there was no relationship (*P*=0.3). We performed a univariate analysis of OS for circulating DNA in the serum using the median concentration (221 ng ml^−1^) as a cut point. The median was chosen since serum DNA is a new factor for analysis and it would give an equal number of samples in each of the two sets. We observed an inverse association where patients with serum DNA concentration >221 ng ml^−1^ had longer survival than patients with serum DNA concentration ⩽221 ng ml^−1^ (*P*=0.028) (presented in Figure 4

). The positive association of serum DNA with better OS seen in the survival curve is endorsed when the set is divided into three equal groups, with cut points 118.5 and 348 (Kaplan–Meier, *P*=0.021; data not shown).

## DISCUSSION

In this study, we measured the level of circulating DNA in the serum of healthy controls and four different groups of patients with breast cancer, using a real-time PCR quantitative method (Lo *et al*, 2000; Leung *et al*, 2001). This real-time detection methodology generates quantitative data during the exponential phase of PCR, has a linear dynamic range of at least five orders of magnitude and enables 96 reactions to be performed in less than 3 h. The need for postamplification processes such as electrophoresis is eliminated.

Our results show a four-fold difference in the median levels of circulating DNA in serum between breast cancer patients and healthy controls (medians of 221 and 63 ng ml^−1^, respectively). Of 96 patients, 69 had DNA concentrations of more than 100 ng ml^−1^ (72%), where only three out of 24 controls had DNA concentrations of more than 100 ng ml^−1^ (12.5%). The ROC curve result (0.92) demonstrates a powerful association between high DNA concentration and malignancy. However, the predictive value of the test depends on prevalence of abnormal cases, and cannot be applied universally. The levels of circulating DNA did not differ between the groups of patients. All the groups showed elevation compared to the controls. The groups of patients were carefully chosen in this pilot study to be able to detect whether there was a difference between patients with high and low bulk of disease, as reflected in node involvement *vs* no node involvement, as well as different tumour sizes reflecting the range from 0.1 to 10 cm. In addition, groups with good or poor prognosis were studied. Choosing distinctive groups did not highlight association between serum DNA concentrations and known prognostic factors.

Other investigators have reported measurements of circulating DNA in cancer, although they have not used quantitative PCR. For example, Sozzi *et al* (2001) quantified circulating DNA in the plasma of 84 patients diagnosed with lung cancer and 43 healthy controls, using the DNA DipStick-TM Kit (Invitrogen). In all, 96% of the patients and 74% of the controls had measurable circulating DNA and the mean value measured was 318 and 18 ng ml^−1^, respectively. High concentrations of circulating DNA were observed from stage Ia of the disease but no correlations were found with age, sex or survival. We believe that since circulating DNA is significantly elevated in cancer patients from the early stages of the disease and in relatively small tumours, such as primary breast tumours, its measurement may prove to have clinical utility in diagnosis and in disease monitoring.

The origin of circulating DNA is not fully understood. In a recent study, Lui *et al* studied the origin of circulating DNA in the setting of sex-mismatched bone marrow transplantation, concluding that the plasma DNA was predominantly of donor origin, that is released from haematopoietic cells (Lui *et al*, 2002). Whether the elevated concentrations of circulating DNA in cancer patients have the same origin or derive from another source of cells, for example the tumour itself, need further investigation. Qualitative studies have shown that genetic alternations such as mutations and LOH were detected in circulating DNA, as well as in the matching tumour cells, suggesting that part of the extra circulating DNA in cancer patients is attributed to DNA released from tumour cells (Gonzalez *et al*, 2000; Shaw *et al*, 2000; Beau-Faller *et al*, 2003). However, our results indicate that the mechanisms of DNA released from tumours are not related to any of the known commonly used major prognostic factors and therefore might highlight different pathways, such as apoptosis, necrosis, hypoxia, that would need to be investigated. It seems likely that at least a proportion of the excess serum DNA in patients with tumours originates from a host response to the tumour (i.e. normal rather than tumour DNA). We found that patients with higher serum DNA concentrations had better overall survival. It may be postulated that higher levels of DNA represents a better host response to the tumour and hence better survival rates. It will be important to develop methods for the separate quantitation of host and tumour DNA.

Prospective studies investigating circulating DNA levels from diagnosis, during and after treatment are necessary to gain insight into their biological behaviour and to asses their clinical utility as disease markers.
